# DNA-Based Assembly of Quantum Dots into Dimers and Helices

**DOI:** 10.3390/nano9030339

**Published:** 2019-03-02

**Authors:** Tao Zhang, Tim Liedl

**Affiliations:** Faculty of Physics and Center for Nanoscience (CeNS), Ludwig-Maximilians-Universität München (LMU), D-80539 Munich, Germany; tim.liedl@physik.lmu.de

**Keywords:** quantum dots, DNA–QDs conjugation, DNA nanotechnology

## Abstract

Owing to their unique optical properties, colloidal quantum dots (QDs) have attracted much attention as versatile fluorescent markers with broad biological and physical applications. On the other hand, DNA-based assembly has proven to be a powerful bottom-up approach to create designer nanoscale objects and to use these objects for the site-directed arrangement of guest components. To achieve good colloidal stability and accurate positioning of QDs on DNA templates, robust QD surface functionalization is crucial. Here, we present a simple and reliable conjugation method for the direct attachment of DNA molecules to QDs. Phosphorothiolated regions of chimera oligonucleotides are attached and incorporated into a ZnS layer freshly growing in situ on QDs that were rendered water soluble with hydrophilic ligands in a prior step. The reaction can be completed in a 2 mL plastic tube without any special equipment. The utility of these DNA-labeled QDs is demonstrated via prototypical assemblies such as QDs dimers with various spacings and chiral helical architectures.

## 1. Introduction

Colloidal semiconductor quantum dots (QDs), in comparison to organic fluorophores, offer several advantages, such as a broader excitation spectra, a narrow and sharply defined emission peak, a longer fluorescence lifetime, orders of magnitude higher photochemical stability, and high resistance to photobleaching [1]. QDs are often considered to be artificial atoms and, as a result of the quantum confinement effect, possess exceptional tunability of their electronic energy levels [2,3,4,5]. Their excitation recombination energies, recombination rates, and the spatial distributions of electrons and holes can be carefully engineered by controlling the size, shape, crystal structure, and composition of the constituent materials. Despite their potential toxicity [6,7] and slow diffusion due to their large physical sizes compared with organic dyes [8], QDs have received considerable attention for various applications in bio-imaging, real-time tracking, and therapeutic drug delivery [9,10,11]. Moreover, their unique optical properties also make QDs appealing candidates for light-emitting diodes, photovoltaic cells, quantum computation, and communications, and they have recently entered the market of consumer electronics [12,13].

To control the photophysical properties of QDs, the primary chemical interest is to master their synthesis to obtain nanocrystals with defined shapes and sizes. As the crystal structure of an individual QD is crucial for its intrinsic properties, a rigorous understanding of the structure–property relationship will allow researchers to optimize QD synthesis and to obtain designed photonic properties in an iterative manner. In fact, ever since the groundbreaking work of growing CdSe nanocrystals in trioctylphosphine oxide (TOPO) was achieved [14], the chemistry at the organic–inorganic interface has been more and more regulated during QD synthesis and has reached exquisite control today. This is achieved by tuning numerous parameters, such as precursors, organic surfactants, solvents, and temperature or through cation exchange reactions [15]. Spherical QDs, one-dimensional (1D) quantum nanorods (NRs), and intricately branched nanocrystals have been successfully synthesized with high uniformity through kinetic shape control [16,17,18,19]. Another way to modify the physical properties of QDs is to organize them in defined geometries where the local electromagnetic environment can be determined by neighboring QDs or by other means, e.g., metallic nanoparticles. Assembled QDs thereby enable a range of studies, such as plasmon–exciton interactions for excitation and emission enhancement, carrier–carrier interactions, and nanoscale spin and energy transfers [20,21,22,23].

To develop practical approaches for precise QD placement, various paths can be followed. Through lithography or slow solvent evaporation, large-scale QD arrays or densely packed QD lattices can be created [24,25]. However, with respect to these processes, there remain challenges to generating finite-sized assemblies containing discrete numbers of components with controlled particle–particle spacings. Alternatively, bottom-up self-assembly positions individual nanoparticles in space via a spontaneous process without the requirement of cumbersome equipment. During self-assembly, the surface ligands, temperature, and ionic strength of the solvent and multiple other parameters define the inter-particle interactions. In principle, any kind of interactive molecular pairs can be implemented to promote nanoparticle assembly, as long as the molecules of interest can be attached to the nanoparticles to thus translate the molecular interactions to the interactions between the coated nanoparticles. Through this approach, higher order nanoparticle assembly has been demonstrated by using ligands such as small molecules [26], polymers [27,28], peptides [29], proteins [30], and DNA [31,32,33]. In particular, due to the simple rule of base pair complementarity and tunable hybridization strength via temperature, solvent conditions (such as salt concentrations, pH, etc.), strand lengths and sequences, DNA allows researchers to introduce highly specific, orthogonal designs of multiple ligands. Furthermore, DNA nanotechnology has the capacity to fold discrete nanostructures of intricate shapes with high yields, thus serving as a perfect template for QD assembly [34].

Generally speaking, in this work, we address a threefold challenge for DNA-based QD assembly: i) to reliably attach DNA to QDs, ii) to obtain designed QD assemblies with high yields, and iii) to characterize optical signatures. Previous endeavors involving DNA–QD conjugation include direct conjugation with thiolated DNA [35], phosphorothiolated DNA oligonucleotides (PTO-DNA) [36,37] or amino-DNA through heterolinkers [38], indirect conjugation via a streptavidin–biotin linkage [39,40], silica or polymer shells providing chemical groups for DNA coupling [41,42,43], and DNA attachment in situ during QD synthesis [44,45,46]. While ligand exchange usually leads to decreased fluorescence efficiencies and a propensity to precipitation, amphiphilic polymer (Figure S1) or protein encapsulation and using silicon shells leads to an additional, often thick, shell on the QDs, which in turn inhibits distance-sensitive energy and information transfer. In contrast, in situ modification realizes the DNA–QD conjugation during QD (CdTe, CdTe/ZnS) growth using thiolated DNA or PTO-DNA as surface capping ligands. The synthesized QDs thus bear the DNA ligands primarily without any intermediate linker, potentially allowing for small particle–particle distances. QDs with such modifications have already been assembled in a site-specific manner, including the formation of heterostructures of gold nanoparticles (AuNPs) with QDs [47]. So far, however, DNA–QD in situ modification has started from ion precursors and thus necessitated the use of a wet chemistry laboratory to accomplish the QD synthesis or shell growth. Here, we report an in situ surface functionalization method to attach PTO-DNA strands onto ZnS-shelled QDs in the presence of zinc ions and 3-mercaptopropionic acid (MPA) ligands. Our method can be performed without any special equipment in a 2 mL plastic tube. We further demonstrate the distance-controlled assembly of QD dimers and chiral helical arrangements on DNA origami templates.

## 2. Materials and Methods

### 2.1. Quantum Dots

MPA-stabilized core/shell quantum dots CdSe/CdS/ZnS (emission peak at 640 nm [48]) and CdSeS/ZnS (emission peak at 540 or 575 nm; Cytodiagnostics, Burlington, ON, Canada) were used as received. The concentration was estimated from the mass concentration. Zinc nitrate, MPA, and other materials were purchased from Sigma Aldrich (Taufkichen, Germany) unless stated otherwise.

### 2.2. DNA

Phosphorothiolated DNA oligonucleotides (PTO-DNA) (sequence: g_9_–T_9_, phosphorothiolated 9 guanine bases and native 9 thymine bases) were purchased from Eurofins Genomics (Ebersberg, Germany). Scaffold p7249 and p7560 were produced in house via phage-infected *Escherichia coli* cell culturing as described elsewhere [49]. Staple strands for DNA origami folding were synthesized by Eurofins Genomics.

### 2.3. DNA–QD Modification

**Figure 1:**
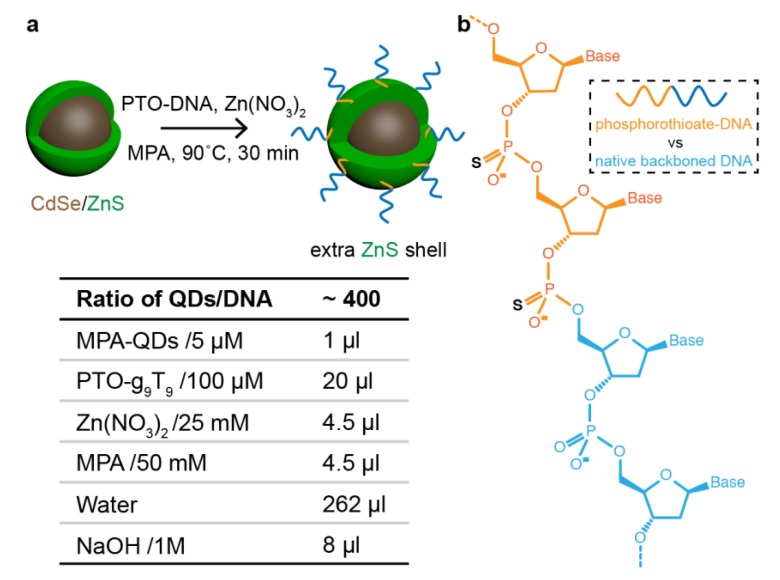
Schematic illustration of incorporating phosphorothiolated DNA oligonucleotides (PTO-DNA) into a freshly-grown ZnS shell. (**a**) PTO-DNA in situ attachment to the extra layers of ZnS shell and a table showing the different precursors; (**b**) Molecular structure of a chimera DNA strand showing two phosphorothiolated backbone bond (orange section) and one native bond (blue section). The black “S” highlights the atomic difference of the upper phosphate link. MPA: 3-mercaptopropionic acid.

The conjugation method was adopted from previous reports (Figure 1) [46,50]. Briefly, 100 µL of 100 nM MPA-capped, ZnS-shelled QDs (in water) were mixed with 20 µL of 100 µM PTO-DNA (sequence g_9_–T_9_, in water), followed by adding 4.5 µL of 25 mM Zn(NO_3_)_2_ (in water) and 4.5 µL of 50 mM MPA (in water). The mixture was gently shaken before adding 4 µL of 1 M NaOH (in water) to deprotonize the MPA ligands. Thereafter, the mixture was incubated at 90 °C for different times. DNA-conjugated QDs were purified using 100 kDa cut-off Amicon filters at a speed of 4600 g for 2 min, followed by an additional 3 rounds of washing by adding 500 µL of water each time, followed by spinning at 4600 g.

### 2.4. DNA Origami

The one-layer sheet and the 24-helix bundle (24-HB, with DNA duplex stacked in a honeycomb lattice) used in this study were designed with caDNAno [51]. DNA staple strands (100 nM each) and the circular DNA scaffold strand (10 nM p7249 for one-layer sheet or 10 nM p7560 for 24-HB) in 1 × TE-Mg^2+^ buffer (10 mM Tris base, 1 mM Ethylenediaminetetraacetic acid (EDTA), 14 mM MgCl_2_) was thermally annealed from 65 to 4 °C over 27 h (15 min at 65 °C, cooling to 58 °C with a cooling rate of −1 °C per 5 min, from 58 to 35 °C with a rate of −1 °C per 1 h, and from 35 to 4 °C with a rate of −1 °C per 5 min). The folded DNA nanostructures were purified from excess DNA staple strands by agarose gel electrophoresis (0.7% agarose in 1 × TAE (40 mM Tris base, 20 mM Acetic acid, 1 mM EDTA), 11 mM MgCl_2_ buffer; 6.5 V/cm for 2 h) stained with 1 × Sybr Safe (Thermo Fisher Scientific, Dreieich, Germany) in ice water baths. The origami samples were extracted from the corresponding gel bands by excision of the bands and recovery of the products by squeezing the gel pieces between two glass slides and collecting the resulting liquid droplet with a pipette.

### 2.5. QD Assembly on DNA Nanostructures

For both dimer and helical assembly, DNA origami together with a 10-time excess of QDs per site were mixed and incubated at room temperature (25 °C) for 6 h. The QD–DNA nanostructure assemblies were purified from excessive QDs and clusters via agarose gel electrophoresis. The gel electrophoresis method was carried out as described above but without staining the gel, as the QDs are bright enough to serve as a reference.

### 2.6. TEM Imaging

Transmission electron microscopy (TEM) imaging of the DNA origami and QD–DNA origami assemblies was carried out using a JEM-1011 transmission electron microscope (JEOL, Tokyo, Japan) operating at 80 kV. Typically, 10 μL of sample volume was deposited onto Argon plasma-treated formvar/carbon-coated (copper mesh) grids (Ted Pella, Inc., Redding, CA, USA; prod no. 01753-f) for up to 1 min. The drop was then removed using filer paper, and the grid was washed and stained with 2% aqueous uranyl formate solution.

## 3. Results

**Figure 2:**
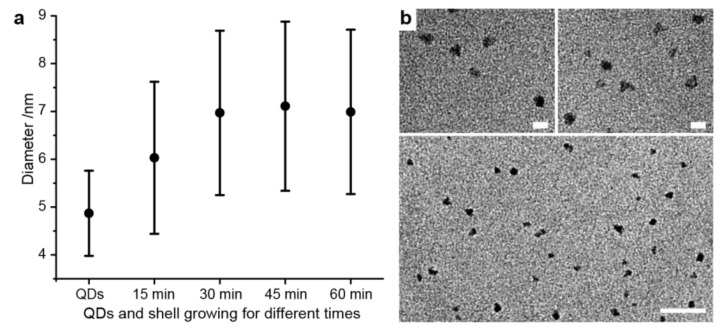
(**a**) Size analysis for quantum dots (QDs) without conjugation shells and QDs incubated with DNA, Zn(NO_3_)_2_, and 3-mercaptopropionic acid (MPA) for different times; (**b**) Transmission electron microscopy images for PTO-DNA-modified CdSeS/ZnS after 30-min incubation at 90 °C. Scale bars: 10 nm (upper panels) and 50 nm (lower panel). TEM images for QDs incubated for different times are included in the supporting information (Figures S2–S4).

We first analyzed the sizes of the CdSeS/ZnS QDs with their additionally grown ZnS shell plus the incorporated DNA strands depending on the incubation time (cf. protocol above). The contrast of the unstained samples was sufficient to allow the measurement of the sizes of the particles. QDs incubated for a longer time showed increased nanoparticle sizes in comparison with the original QDs due to the newly grown ZnS shell (Figure 2a). While the QDs grown for 15 min have a diameter of 6.0 ± 1.6 nm (standard deviation), all the other samples are larger. These results agree well with a previous report [52]. Interestingly, the average sizes of the particles after an incubation time of 30, 45, and 60 min are very similar (7.0 ± 1.8 nm, 7.1 ± 1.8 nm, and 7.0 ± 1.7 nm, respectively). This could be a result of the depletion of the growing materials and a completion for the DNA attachment. Figure 2b shows TEM images of dispersed DNA-conjugated QDs incubated at 90 °C for 30 min in water. More TEM images and analytical methods are included in the supporting information (Figures S2–S4). Agarose gel electrophoresis (Figure S5) reveals that only DNA-conjugated particles enter the gel. With increasing incubation time, a decreased brightness of the corresponding bands is observed, indicating the reduction of the optical stability of the QDs.

**Figure 3:**
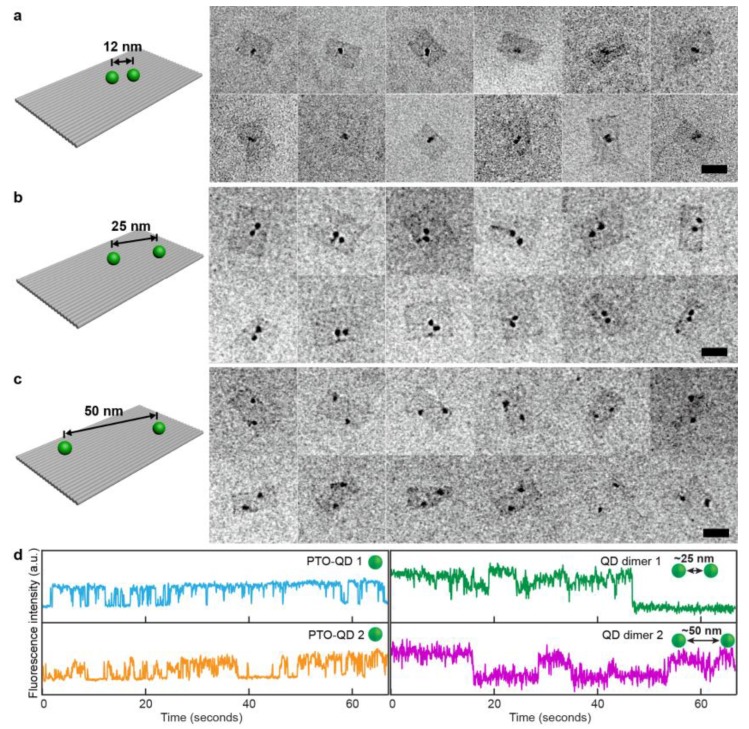
Assembly scheme and corresponding transmission electron microscopy images of CdSe/CdS/ZnS quantum dots assembled into dimers with different distances on one-layer rectangular DNA origami templates. (**a**) QD dimer with 12 nm inter-particle spacing; (**b**) 25 nm inter-particle spacing; (**c**) 50 nm inter-particle spacing. Scale bars: 50 nm; (**d**) Fluorescence intensity traces of various QD samples: individual PTO-DNA modified QDs (blue and orange), QD dimers with 25 nm (green) and 50 nm (purple) spacings.

We then tested the assembly of DNA-conjugated QDs on DNA structures. We first prepared dimers of CdSe/CdS/ZnS QDs with various spacings on a one-layer DNA origami template. As shown in Figure 3a–c, the QDs assembled in dimers with designed spacings of 12, 25, and 50 nm (Figure S6). In the TEM images, the rectangular DNA origami templates are clearly visible despite the low contrast provided by the single layer of double-stranded DNA. The darker spots indicate the QDs, which were located at the designated sites and were individually distinguishable in the cases of the larger particle distances. The average spacing was measured to be 12 ± 5 nm, 24 ± 7 nm, and 53 ± 9 nm, respectively. The large variations are a result of occasional bending and twisting of the single-layered DNA origami templates, which lack rigidity [53]. We found that the dimer assembly yield critically depends on the distance between the QDs. Due to the increased steric and electrostatic repulsion forces between the particles when forced close to each other, the binding efficiency of the dimer structures was found to be ~66% for the 50 nm spacing, ~56% for the 25 nm samples, and only ~30% for the 12 nm spacing. The attachment yield per site reached a yield of 82% per site, which is below that of previously reported methods [40,46], due to the following disadvantages. Firstly, particles sometimes bind non-specifically to the scaffold loops that connect the neighboring helices at their ends, resulting in trimer assemblies. Another reason for the relatively low attachment yields—in particular if compared with gold particle attachment yields, which can reach 99% [54]—is the formation of QD clusters in the presence of magnesium ions. As mentioned above, for the short distances, it was difficult to distinguish the individual QDs, especially after the staining of the samples with uranyl materials. The numbers are given for CdSe/CdS/ZnS QDs. Other materials or different core/shell designs of the QDs can further influence the assembly yield.

To determine the influences of surface modification and DNA origami assembly on the optical properties of the QDs, blinking behavior was studied using a fluorescence microscope (Olympus, Tokyo, Japan). The QD samples were spread on glass slides and fluorescence movies were collected with an iXon EMCCD camera (Andor Technology, Belfast, UK) from dried samples. For both, individual DNA-modified QDs and QDs dimer assemblies, switching between bright (ON) and dark (OFF) states on multiple time scales was observed, which indicates typical behavior of fluorescent quantum dots [55] (Figure 3d and Figures S7 and S8). Dimers also exhibited the expected three-state behavior; however, the time traces of the QD dimers showed such fast bleaching (within several tens of seconds and less) that further analysis of the traces, e.g., testing the influence of the inter-particle distance on the blinking rates, yielded ambiguous results. This result indicates a significant loss of optical stability after the assembly and purification processes and is in line with the observation of the reduced brightness of the particles after conjugation (Figure S5).

**Figure 4:**
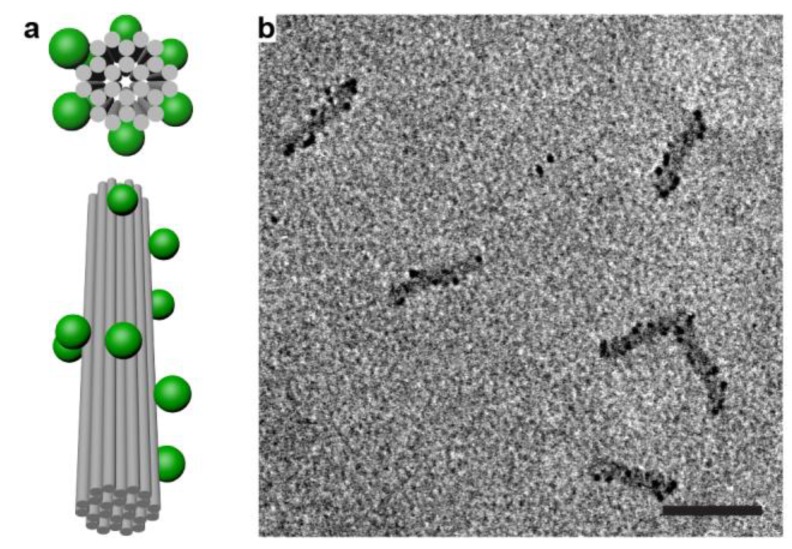
QDs assembled into chiral (here left-handed) helices. (**a**) Top and side view of the chiral QD assemblies on a 24-HB DNA origami; (**b**) TEM image of unstained 24-HB with helical assemblies of CdSeS/ZnS particles. Scale bar: 100 nm.

Finally, we applied our method to build higher order QD assemblies. Figure 4 shows the chiral arrangement of fluorescent colloidal QDs on a 24-HB (Figure S9), which were here left-handed helices, which in principle could give rise to polarization specific absorption and emission. We did not observe these effects, which we attribute to the still relatively large distance between the particles (the designed center-to-center distance was ~11 nm [54]) and the low concentration (below 50 pM) of our assembled helices. Nevertheless, such systems could open new perspectives for luminescence-based detection and sensing.

## 4. Conclusions

With our simple method, we realized stable DNA functionalization of ZnS-shelled QDs. By attaching PTO-DNA to a newly-grown ZnS shell, a simple conjugation is established that ensures solubility even at high Mg^2+^ or Na^+^ concentrations, allowing for site-specific positioning of these QDs on DNA origami templates. To recapitulate the threefold challenge mentioned above—reliable DNA conjugation to QDs, spatial assembly of these QDs, and optical characterization—we here offered new and simple ways to overcome the first challenge. We also obtained satisfactory assembly yields of particle dimers with defined distances and helical QD arrangements (overcoming the second challenge). To improve optical signals that would allow for optical analysis, several issues need to be addressed in future research. For one, the success of QD stabilization may critically depend on the used DNA sequence that interacts with the surface of the QDs. Also, the integration of functional chemistry groups, e.g., azides, alkynes, or multidentate thiol ligands, into the DNA strands could protect the QDs effectively through robust chemistry. To then observe the intricate behavior of coupled quantum emitter systems, QDs with outstanding optical properties will be required. Ultimately, DNA-based QD assembly in complex geometries will offer the possibility to probe fundamental interactions, such as exciton–exciton interactions or, for example, frustrated spin systems [56,57,58,59,60,61,62]. 

## Supplementary Materials

The following figures are available online at https://www.mdpi.com/2079-4991/9/3/339/s1, Figure S1: Transmission electron microscopy (TEM) images for CdSe/CdS/ZnS quantum dots (QDs) encapsulated in amphiphilic polymers, Figure S2: TEM images for PTO-DNA functionalized CdSeS/ZnS QDs incubated for different times, Figure S3: QD size distribution analysis with ImageJ, Figure S4: Size distribution of QDs and QDs incubated for different times, Figure S5: Agarose gel electrophoresis of QDs incubated for different times, Figure S6: TEM images for CdSe/CdS/ZnS QD dimer assemblies (spacing 50 nm) on a one-layer rectangular DNA origami template before purification, Figure S7: Fluorescence intensity time trace of 10 different PTO-DNA-functionalized CdSe/CdS/ZnS QDs, Figure S8: Fluorescence intensity time trace of CdSe/CdS/ZnS QD dimers on a one-layer sheet DNA origami with two separations, 25 and 50 nm, and Figure S9: Agarose gel electrophoresis of CdSeS/ZnS QDs (emission at 540 and 575 nm) chiral assemblies on 24-HB origami.
