# Supplementary material for: DNA-Based Assembly of Quantum Dots into Dimers and Helices
Source: Nanomaterials (Basel). 2019 Mar 2;9(3):339. doi: 10.3390/nano9030339 (PMC6473979; doi:10.3390/nano9030339)
Supplement: Supplementary file 1 [file nanomaterials-09-00339-s001.pdf]

Supplementary Information
DNA-Based Assembly of Quantum Dots into Dimers and Helices

Tao Zhang*,†,‡ and Tim Liedl†

†*Faculty of Physics and Center for Nanoscience (CeNS), Ludwig-Maximilians-Universität München (LMU), D-80539 Munich, Germany*

‡*Current address: Max-Planck-Institute for Intelligent Systems, D-70569 Stuttgart, Germany*

E-mail: tao.zhang@is.mpg.de

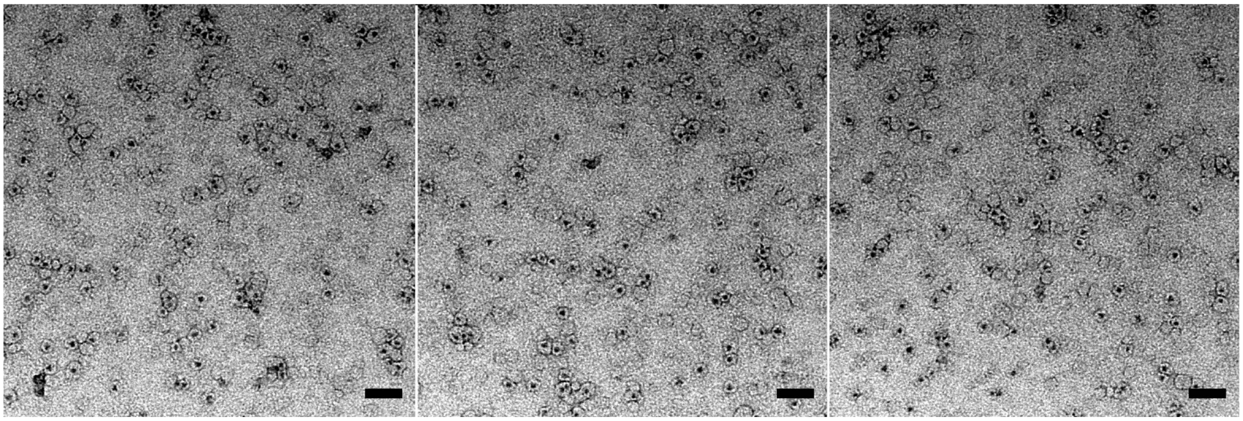

Figure S1: Transmission electron microscopy (TEM) images for CdSe/CdS/ZnS quantum dots (QDs) encapsulated in amphiphilic polymers (protocols adopted from previous publications[1,2] and experimental details can be found in Tao Zhang's PhD thesis[3]). Grids were stained with 2% aqueous uranyl formate solution. Scale bar: 50 nm.

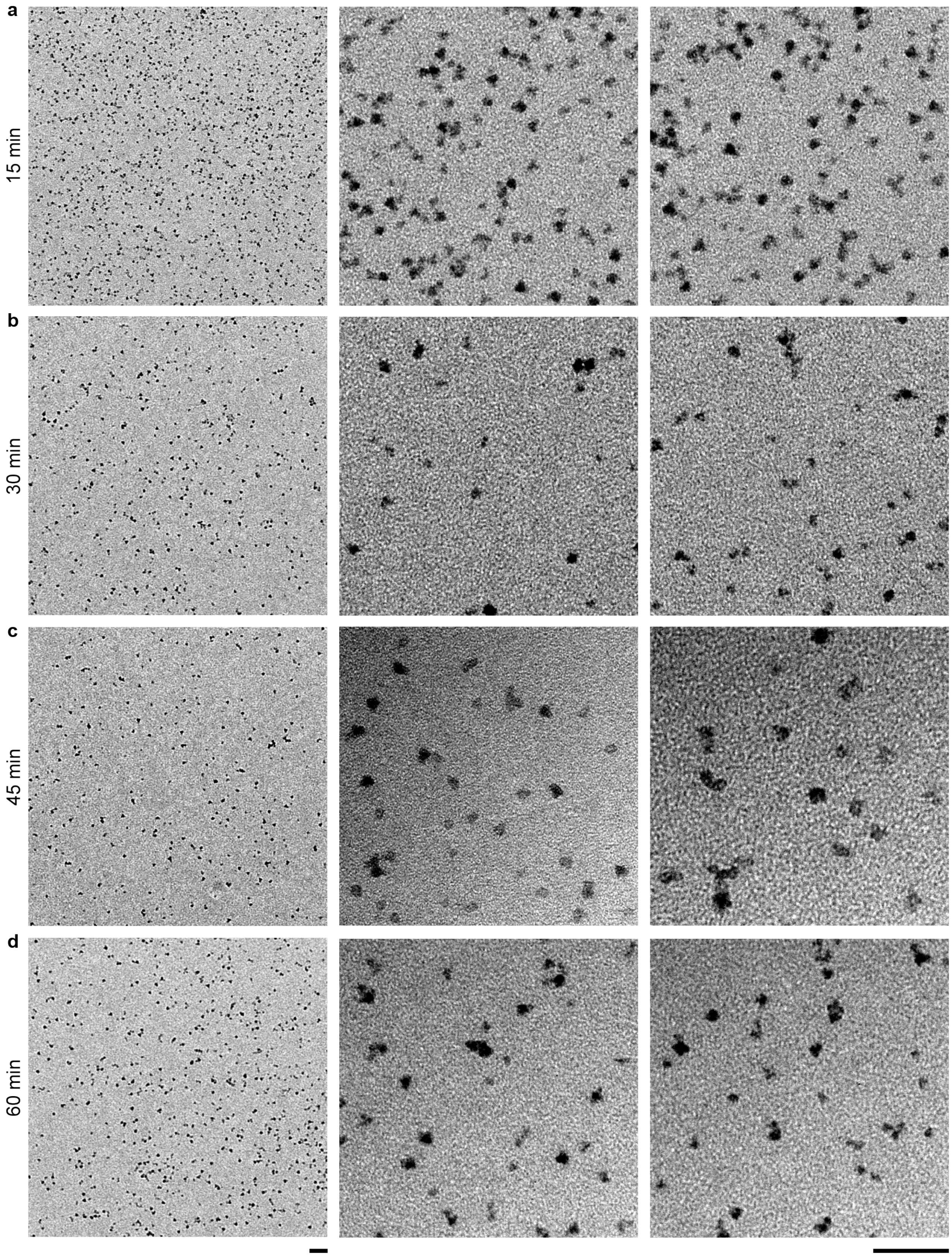

Figure S2: TEM images for PTO-DNA functionalized CdSeS/ZnS QDs incubated for different times. Scale bar: 50 nm.

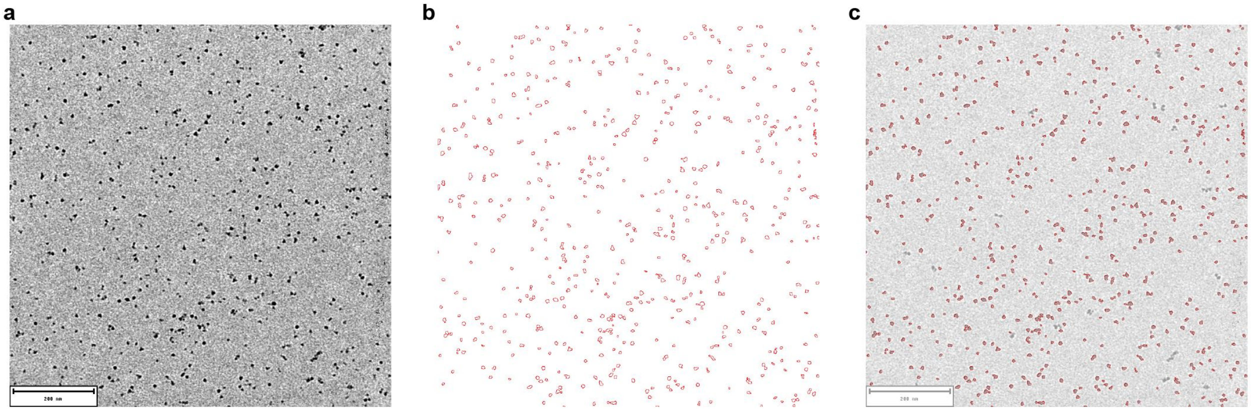

Figure S3: QD size distribution analysis with ImageJ. The nanoparticles size distribution was determined automatically with image processing program ImageJ. The protocol is, 1) calibrate the image magnification, 2) find the threshold for the image, and 3) analyse the nanoparticles sizes. Panel a shows a TEM image of CdSeS/ZnS incubated for 30 mins, panel b shows the image with outlined nanoparticles after manually determined threshold, and panel c shows the superpositioned images in panel a and b. Online protocol can be found at https://imagej.net/Particle_Analysis (visited on 31st Jan. 2019).

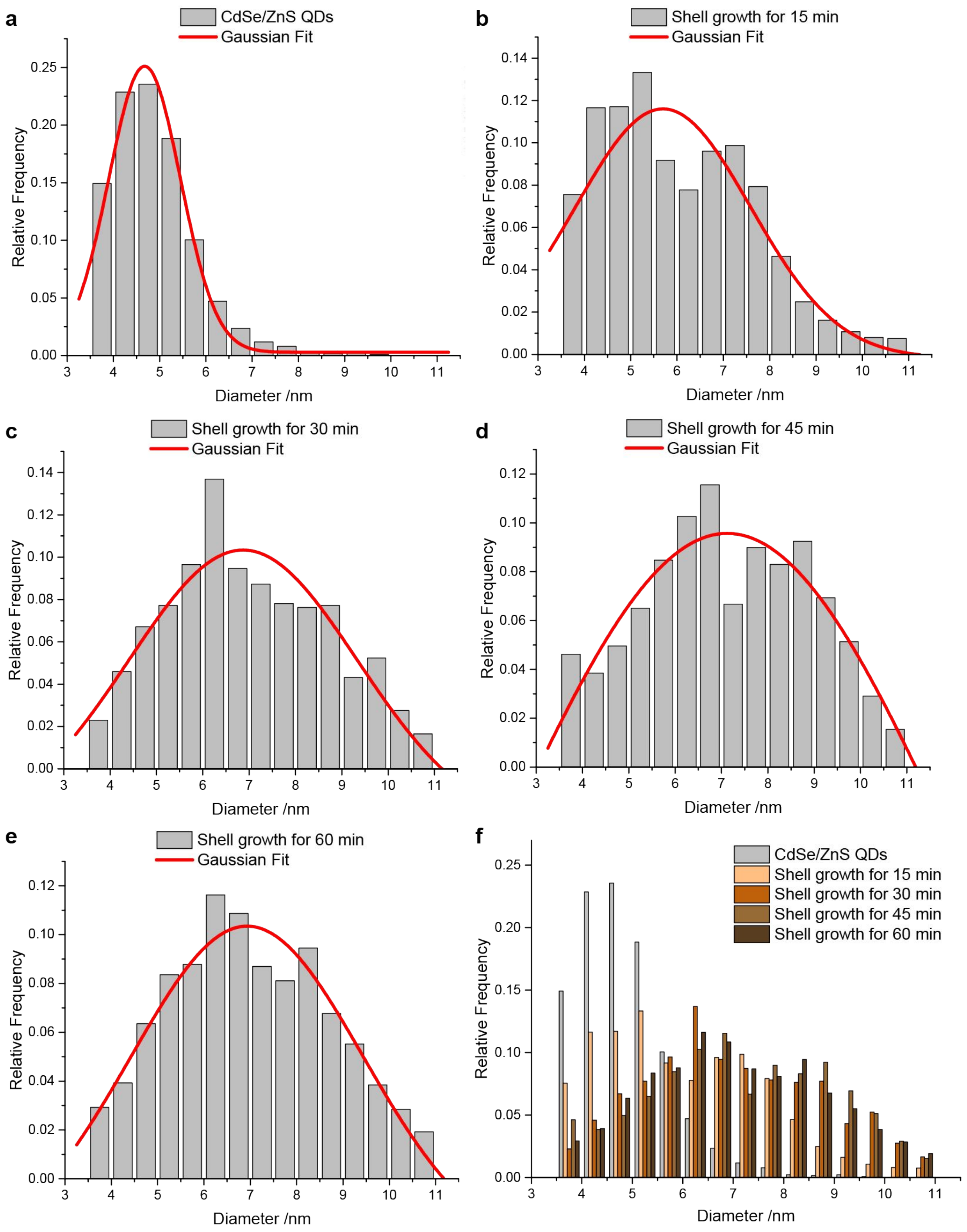

Figure S4: Size distribution of QDs and QDs incubated for different times. Panel f shows a full comparison of all the particles.

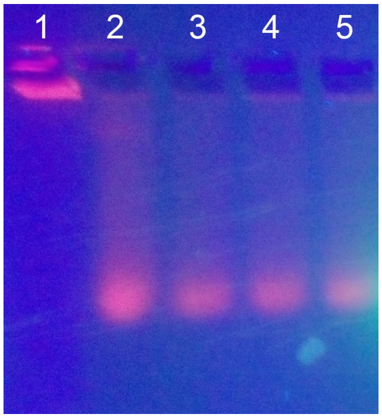

Figure S5: Agarose gel electrophoresis of QDs incubated for different times. Lane 1: the control sample of QDs (Cytodiagnostics, emission peak at 630 nm) before DNA functionalization, Lane 2-5: QDs incubated with PTO-DNA, zinc nitrate and 3-mercaptopropionic acid for 15 min, 30 mins, 45 mins, and 60 mins, respectively. The PTO-DNA functionalized QDs showed decreased brightness along with the incubation time because of the loss of the photonic stability. The delayed mobility of PTO-DNA functionalized QDs is attributed to the increased loading of DNA on QDs with increasing incubation time. Gel electrophoresis conditions: 0.7% agarose; 1x TAE buffer; 6.5 V/cm for 1 h.

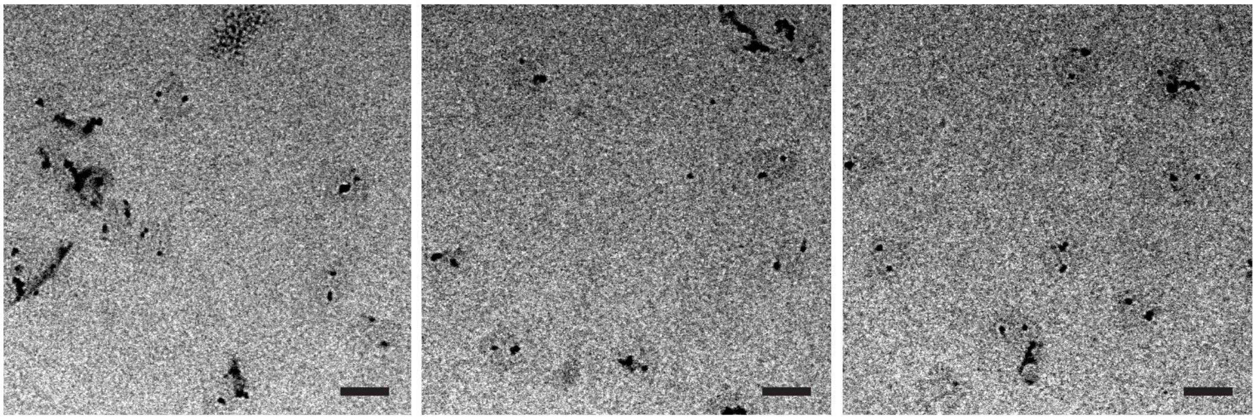

Figure S6: TEM images for CdSe/CdS/ZnS QD dimer assemblies (spacing 50 nm) on a one-layer rectangular DNA origami template before purification. Scale bars: 100 nm.

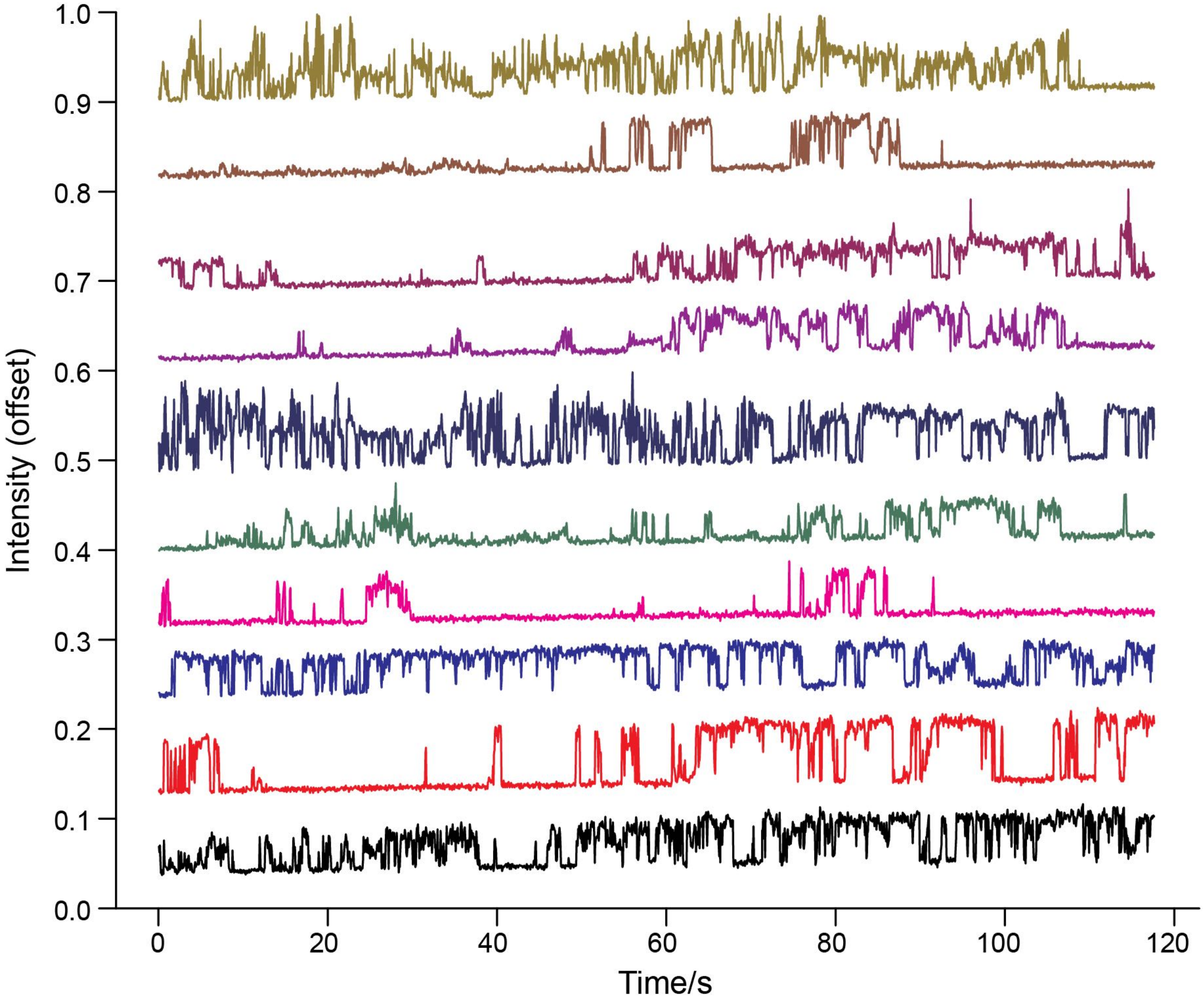

Figure S7: Fluorescence intensity time trace of 10 different PTO-DNA functionalized CdSe/CdS/ZnS QDs. In the time range of 2 minutes, multiple ON (bright) and OFF (dark) states were observed. The ON and OFF rates are different from dot to dot, showing the diversities of the optical properties for QDs.

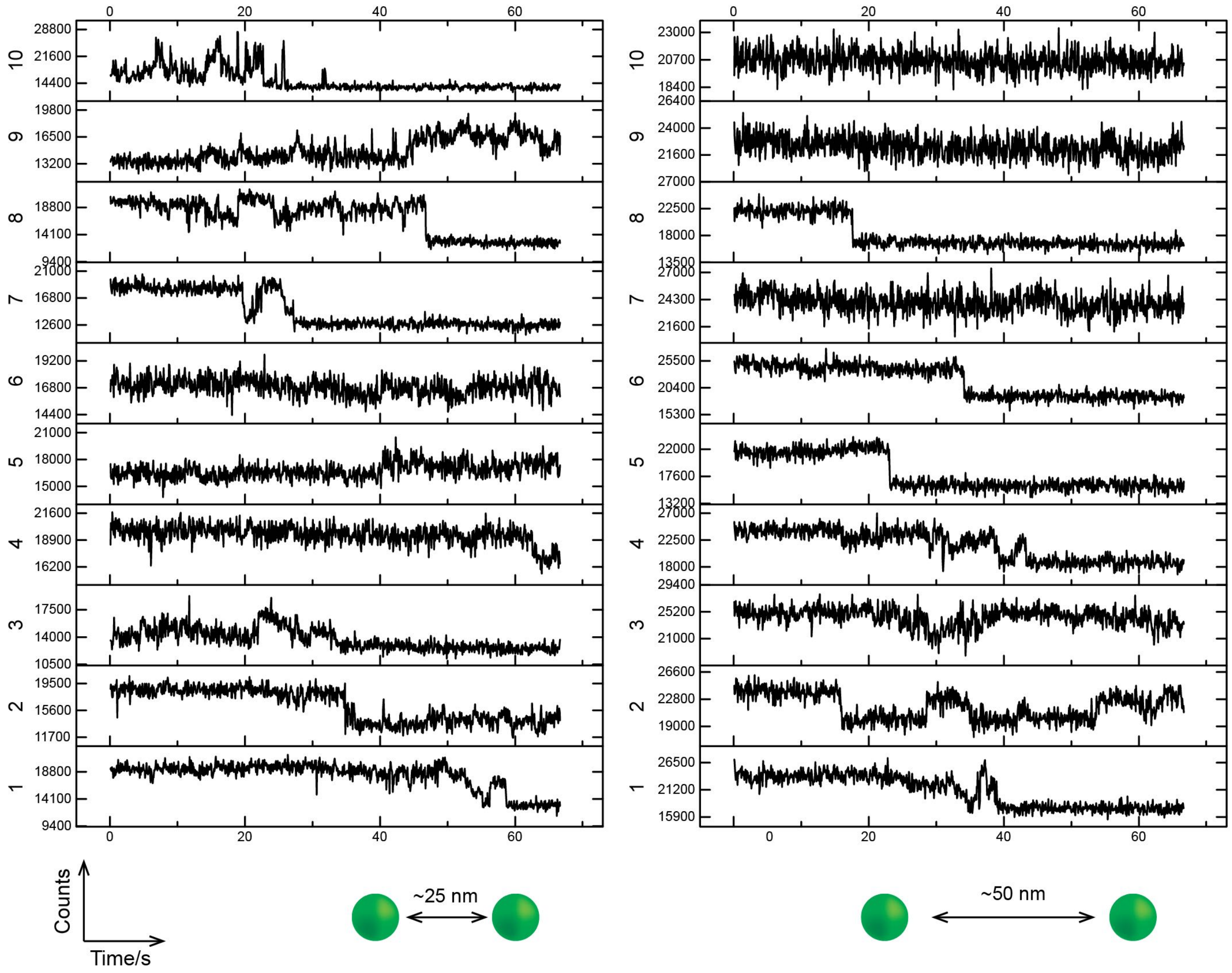

Figure S8: Fluorescence intensity time trace of CdSe/CdS/ZnS QD dimers on a one-layer sheet DNA origami with two separations, 25 and 50 nm. Due to subdiffraction-spaced assemblies, the blinking states from individual quantum dot are not resolved.

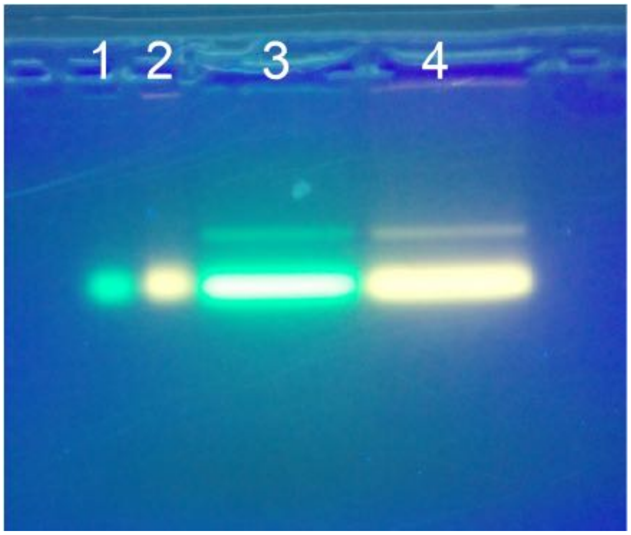

Figure S9: Agarose gel electrophoresis of CdSeS/ZnS QDs (emission at 540 nm and 575 nm) chiral assemblies on 24-HB origami. Lane 1 & 2, PTO-DNA functionalized QD with emission at 540 nm and 575 nm respectively. Lane 3 & 4, the DNA functionzlied QDs chiral assemblies on 24-HB DNA origami correspondingly to the QDs in lane 1 & 2. In lane 3 & 4, the lower bands are the excessive QDs, while the upper ones are the QDs chiral assemblies on 24-HB. Gel electrophoresis conditions: 0.7 % agarose gel; 1x TAE buffer; 11 mM $MgCl_2$ buffer; 6.5 V/cm for 1 hour.

References

(1) Lin, C.-A. J.; Sperling, R. A.; Li, J. K.; Yang, T.-Y.; Li, P.-Y.; Zanella, M.; Chang, W. H.; Parak, W. J. Design of an amphiphilic polymer for nanoparticle coating and functionalization. *Small* **2008**, *4*, 334–341.

(2) Pellegrino, T.; Manna, L.; Kudera, S.; Liedl, T.; Koktysh, D.; Rogach, A. L.; Keller, S.; Rädler, J.; Natile, G.; Parak, W. J. Hydrophobic nanocrystals coated with an amphiphilic polymer shell: a general route to water soluble nanocrystals. *Nano Lett.* **2004**, *4*, 703–707.

(3) Zhang, T. Assembly of optically-active nanocomponents with DNA. Ph.D. thesis, Ludwig Maximilian University of Munich, 2017.
